# A combinatorial DNA assembly approach to biosynthesis of *N-*linked glycans in *E. coli*

**DOI:** 10.1093/glycob/cwac082

**Published:** 2023-01-13

**Authors:** Ian J Passmore, Alexandra Faulds-Pain, Sherif Abouelhadid, Mark A Harrison, Catherine L Hall, Paul Hitchen, Anne Dell, John T Heap, Brendan W Wren

**Affiliations:** London School of Hygiene & Tropical Medicine, Department of Infection Biology, London, WC1E 7HT, UK; University of Nottingham, School of Life Sciences, Nottingham, NG7 2RD, UK; London School of Hygiene & Tropical Medicine, Department of Infection Biology, London, WC1E 7HT, UK; London School of Hygiene & Tropical Medicine, Department of Infection Biology, London, WC1E 7HT, UK; London School of Hygiene & Tropical Medicine, Department of Infection Biology, London, WC1E 7HT, UK; Imperial College London, Department of Life Sciences, London, SW7 2AZ, UK; Imperial College London, Department of Life Sciences, London, SW7 2AZ, UK; University of Nottingham, School of Life Sciences, Nottingham, NG7 2RD, UK; London School of Hygiene & Tropical Medicine, Department of Infection Biology, London, WC1E 7HT, UK

**Keywords:** bioconjugation, DNA assembly, glycoconjugate vaccines, N-glycosylation, synthetic biology

## Abstract

Glycoengineering of recombinant glycans and glycoconjugates is a rapidly evolving field. However, the production and exploitation of glycans has lagged behind that of proteins and nucleic acids. Biosynthetic glycoconjugate production requires the coordinated cooperation of three key components within a bacterial cell: a substrate protein, a coupling oligosaccharyltransferase, and a glycan biosynthesis locus. While the acceptor protein and oligosaccharyltransferase are the products of single genes, the glycan is a product of a multigene metabolic pathway. Typically, the glycan biosynthesis locus is cloned and transferred en bloc from the native organism to a suitable *Escherichia coli* strain. However, gene expression within these pathways has been optimized by natural selection in the native host and is unlikely to be optimal for heterologous production in an unrelated organism. In recent years, synthetic biology has addressed the challenges in heterologous expression of multigene systems by deconstructing these pathways and rebuilding them from the bottom up. The use of DNA assembly methods allows the convenient assembly of such pathways by combining defined parts with the requisite coding sequences in a single step. In this study, we apply combinatorial assembly to the heterologous biosynthesis of the *Campylobacter jejuni*  *N*-glycosylation (*pgl*) pathway in *E. coli.* We engineered reconstructed biosynthesis clusters that faithfully reproduced the *C. jejuni* heptasaccharide glycan. Furthermore, following a single round of combinatorial assembly and screening, we identified pathway clones that outperform glycan and glycoconjugate production of the native unmodified *pgl* cluster. This platform offers a flexible method for optimal engineering of glycan structures in *E. coli*.

## Introduction

Heterologous biosynthesis of complex glycan structures involves the cloning and expression of multiple genes that encode the enzymes necessary for their assembly. Bacteria are ideal hosts for this process, known as glycoengineering, as they tolerate manipulation of glycosylation and other glycostructural pathways such as *N*-linked and *O*-linked protein glycosylation systems, capsular polysaccharide, and lipooligosaccharide (LPS) biosynthesis. These recombinant glycans can be covalently linked in vivo to other macromolecules, such as proteins and lipids (Chen et al. 2016; Gerritzen et al. 2017; Valguarnera and Feldman 2017), to generate glycoconjugate vaccines, therapeutics, and diagnostics. This burgeoning technology, known as Protein Glycan Coupling Technology (PGCT), offers the potential to custom design and combine protein and glycan structures in safe, genetically tractable bacterial strains (Kay et al. 2019). Due to their typical surface location and central role in disease, glycans are the prime candidates for diagnostic and glycoconjugate vaccine development (Ladhani 2012; Perrett et al. 2013; Micoli et al. 2018).

Traditionally, PGCT involves transforming *Escherichia coli* cells with three plasmids that encode the requisite components for glycoconjugate production: a substrate protein, an oligosaccharyltransferase, and a glycan biosynthesis locus. The typical approach in the PGCT vaccine field is to attempt to transfer a complete, unmodified glycan biosynthetic gene cluster from a native host into *E. coli* (Cuccui et al. 2013; Kay et al. 2016; Harding et al. 2019; Duke et al. 2021). In some cases, this results in functional, although usually not optimal, glycan biosynthesis. Transcription and translation of genes within these clusters are finely tuned for synthesis in the native host, and simply introducing the unmodified cluster into *E. coli* often results in suboptimal (or no) glycan production. Introduction of a heterologous biosynthetic pathway may be poorly tolerated, or tolerated at the expense of glycoconjugate yield (Glasscock et al. 2018). Furthermore, faithful cloning of large (frequently >15 Kbp) biosynthetic loci as a single contiguous fragment is technically challenging and the incoming pathway may conflict with the endogenous sugar metabolic pathways of the host strain (Ceroni et al. 2015).

A number of advancements have been made to improve the capabilities of PGCT and the yield of the desired glycoconjugate, including (but not limited to) the identification of custom acceptor proteins with improved immunogenicity (Reglinski et al. 2018), identification of novel oligosaccharyltransferases with alternative sugar substrate specificity (Ihssen et al. 2012; Feldman et al. 2019; Harding et al. 2019), and removal of conflicting sugar biosynthetic pathways from the *E. coli* host strain (Yates et al. 2019). However, the glycan biosynthesis pathway is frequently overlooked. To date, the vast majority of advancements to specifically improve glycan yield have focused on the modification of the *E. coli* host strain or the introduction of one or two specific genes rather than targeting the incoming glycan biosynthetic locus itself (Yates et al. 2019; Kay et al. 2022).

In recent years, synthetic biology has tackled the challenge of heterologous expression of complex multigene systems and has fundamentally changed the state of the art. Seminal work introduced “refactoring”: deconstructing complex gene clusters and rebuilding them from the bottom up in a modular format (Temme et al. 2012; Smanski et al. 2014). Combinatorial construction using well-defined parts (synthetic promoters, ribosome-binding sites [RBSs], coding sequences [CDSs], and terminators) has been enabled by new DNA assembly methods that combine multiple DNA parts in a single step (Zelcbuch et al. 2013; Smanski et al. 2014; Awan et al. 2017). The modular and hierarchical nature of these assemblies obviates the need to clone large genetic loci as a single contiguous fragment, facilitates the identification of any components causing issues, and provides a platform for the efficient systematic optimisation of expression and function through iterative cycles of engineering. To date, this approach has not been applied to the biosynthesis of complex glycan pathways.

In this study, our aim was to apply these modern synthetic biology principles to the refactoring and assembly of the *Campylobacter jejuni N-*linked protein glycosylation (*pgl*) biosynthesis pathway in *E. coli*. We refactored the *C. jejuni pgl* cluster by removing each gene from its native regulation and rebuilt it from the bottom up using combinatorial Start-Stop Assembly, a scarless modular DNA assembly system that is well-suited to the construction of combinatorial libraries of multigene expression constructs (Taylor et al. 2019). Each of the 10 CDSs within the *pgl* cluster responsible for biosynthesis of the glycan were assembled with mixtures of synthetic promoters and RBSs of various strengths to generate a large library of expression constructs. We established a rapid screen which, despite only being able to cover a tiny fraction of the library, identified *pgl* clones that outperformed glycan production compared with the native, unmodified *pgl* cluster cloned directly from *C. jejuni*. Furthermore, the improved glycan production translated to a greater glycoconjugate yield when these refactored loci were transformed into glycoengineering strains expressing the *C. jejuni N*-oligosaccharyltransferase, *pglB,* and a target protein harboring an appropriate sequon. Thus, we demonstrate the potential of this approach for improved, refactored sugar biosynthesis loci construction towards recombinant glycan biosynthesis and subsequent glycoconjugate vaccine production.

## Results

### Design and construction of a refactored *C. jejuni pgl* locus expression library

The first bacterial *N*-linked glycosylation system was identified in the human enteric pathogen *C. jejuni* (Szymanski et al. 1999; Wacker et al. 2002)*.* Genetic and biochemical studies revealed that *C. jejuni* modifies at least 50 proteins post-translation with the heptasaccharide, GalNAc-α1,4-GalNAc-[Glcβ1,3-]GalNAc-α1,4-GalNAc-α1,4-GalNAc-α1,3-diBacNAc-β1 (where GalNAc is *N-*acetylgalactosamine, Glc is glucose, and diNacBAc is 2,4-diacetamido-2,4,6-trideoxyglucopyranose) (Linton et al. 2002). The discovery that the *C. jejuni pgl* cluster is fully functional when transferred to *E. coli* unlocked a new area of bacterial glycoengineering and the potential to construct recombinant glycans and glycoconjugates (Wacker et al. 2002). Subsequent discoveries demonstrated that *pglB*, which encodes the oligosaccharyltransferase within this locus, has a relaxed glycan substrate specificity and can transfer a range of glycostructures to almost any protein, provided it possesses the D/E-XNX-S/T sequon (Feldman et al. 2005; Kowarik et al. 2006).

**Fig. 1:**
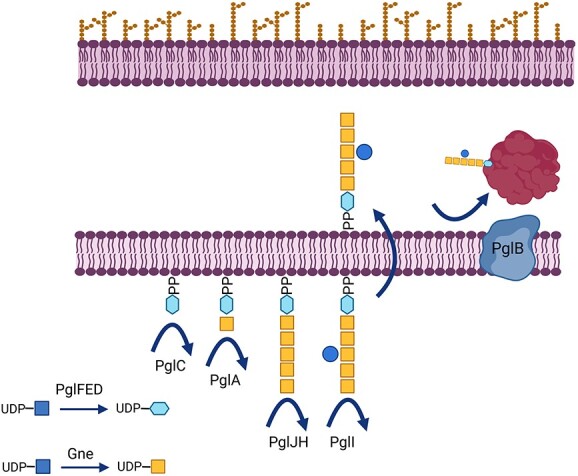
Schematic of the N-glycosylation pathway of *C. jejuni.* Blue squares: GlcNAc, blue hexagon, diNAcBac; yellow squares, GalNAc; and blue circle, Glc; OM, outer membrane; IM, inner membrane.

In this pathway, the enzymes PglFED are responsible for the synthesis of the reducing end sugar UDP-diNAcBac; Gne (alternatively referred to as GalE) is an UDP-glucose 4-epimerase, which catalyzes the conversion of UDP-GlcNAc to UDP-GalNAc; the glycosyltransferase enzymes PglC, PglA, PglJH, and PglI progressively build the heptasaccharide on the lipid carrier undecaprenylpyrophosphate (UndP) on the cytoplasmic side of the inner membrane. This glycan structure is then flipped to the periplasmic side of the membrane by the flippase, PglK. Finally, the oligosaccharyltransferase, PglB, transfers the glycan “en bloc” to asparagine residues within substrate proteins that contain the acceptor sequon D/E-XNX-S/T, where X can be any amino acid except proline (Fig. 1; Linton et al. 2005).

**Fig. 2:**
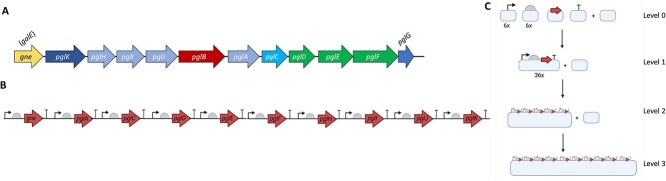
A) Operon architecture of the *pgl* cluster from *C. jejuni* strain 81116. B) Design of the refactored *pgl* pathway with monocistronic architecture. C) Schematic showing Start-Stop assembly for the refactored *pgl* pathway library, which is composed of 10 genes with a standard set of set of 6 promoters, 6 RBSs, and 1 terminator. Plasmids are represented by grey boxes.

The 10 genes necessary for heptasaccharide formation in *E. coli*, *gne*, *pglA*, *pglC*, *pglD*, *pglE*, *pglF*, *pglH*, *pglI*, *pglJ*, and *pglK* (Fig. 2A and B) were selected for pathway refactoring. The CDS for the oligosaccharyltransferase PglB (Figs. 1 and 2A) was deliberately excluded as it would offer more flexibility as to which transferase could be used for coupling to an acceptor protein. Furthermore, the notion that the expression of *pglB* could be detrimental to the host cell has been previously reported (Abouelhadid et al. 2021), and we treated it as a separate engineering challenge for the purpose of this study. Each CDS was searched for BsaI, SapI, and BbsI restriction sites, and those genes which did not contain these restriction sites were amplified by PCR from *C. jejuni* genomic DNA. Those genes that did contain these restriction sites were chemically synthesized, with the restriction sequences removed.

Each CDS within the *pgl* locus was cloned alone, without promoter, RBS, or terminator, into the level 0 Start-Stop Assembly storage vector, pStA0. These were subsequently assembled into transcription units using a standard set of 6 promoters, 6 RBSs, and a single transcriptional terminator, as shown in Fig. 2B and C. Here, equimolar mixtures of the promoter and RBS parts were combined in one-pot assembly reactions with each CDS to vary the expression of each gene in the pathway, producing 36 different expression strengths of each CDS. Blue/white screening was utilized to identify plasmids with insertions (white colonies), and all successfully assembled transformants were pooled and were used to prepare plasmid DNA. These level 1 transcription units were subsequently used to build two 5-gene pathways in level 2 vectors, pStA2, which were then combined into the level 3 vector, pStA3, containing all 10 genes required to produce the *C. jejuni* heptasaccharide (Fig. 2C). Plasmids containing refactored *pgl* libraries were transformed into NEB 10-β, which was exploited for its high transformation efficiency, particularly with high molecular weight plasmids and blue/white screening. Libraries were not further refined, and therefore, as each CDS could be expressed at up to 36 expression levels, and with 10 genes in the pathway, the library had a potential size of (6 × 6)^10^ = 3.6^15^. This number is too large to screen exhaustively but instead provides a diverse pool of variants which can be sampled.

### Screening assays identify successfully refactored sugar synthesis clones from a library of millions

To identify the clones in the refactored *pgl* expression library which were able to synthesize the *C. jejuni* heptasaccharide in *E. coli*, a screen was developed in which the glycan displayed on the surface of the cell was detected by immunoblot. Bacterial protein *N*-linked glycosylation (as utilized by the *C. jejuni pgl* locus) and O-antigen polysaccharide biosynthesis broadly share a similar assembly mechanism in which the glycans are assembled on a lipid carrier on the cytoplasmic face of the inner membrane and are then flipped to the periplasmic face. Once in the periplasm, the glycan can serve as a substrate for both a protein oligosaccharyltransferase (PglB) or the O-antigen ligase, WaaL, which traffic it to a substrate protein or the cell surface, respectively. Here, the overlapping assembly mechanisms were exploited (as they have been previously; Price et al. 2016; Strutton et al. 2019; Yates et al. 2019; Mauri et al. 2021) to harness the native WaaL and attach the preassembled *C. jejuni* heptasaccharide to the outer core of the LPS.

**Fig. 3:**
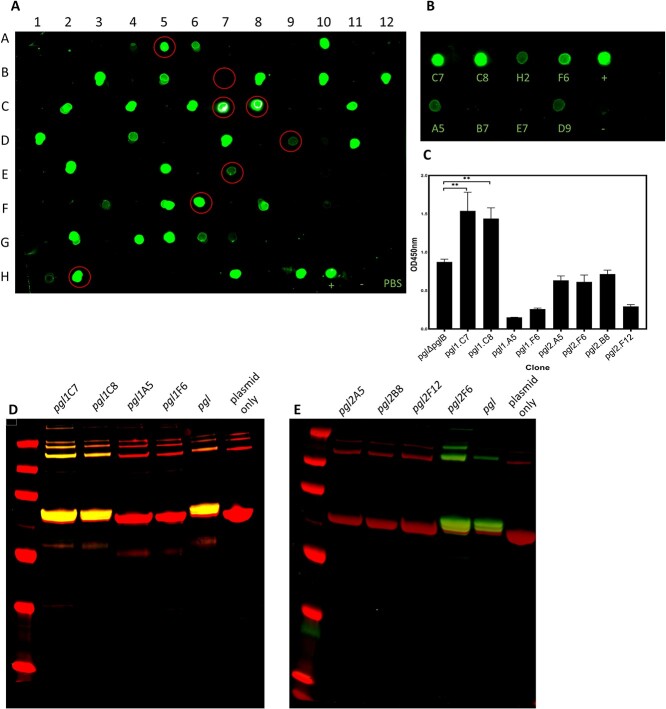
A) Randomly picked clones probed for production of the *C. jejuni* heptasaccharide using an SBA, a biotinylated GalNAc-specific lectin, and green streptavidin-coupled IRDye. Clones were selected at random, grown in high throughput 200-μL cultures, and spotted on nitrocellulose membrane. Clones selected for further characterization are circled in red. B) Clones were subsequently grown in 10-mL cultures, normalised to the same OD, and probed for GalNAc production with SBA. Cells harboring plasmid-borne native unmodified *pgl* cluster and empty plasmid control are denoted as “+” and “-,” respectively. C) ELISA glycan quantification shows a range of glycan production from refactored clusters compared with the wildtype cluster. D and E) Glycoconjugate produced by 8 refactored *pgl* cluster variants from 2 independent assemblies and transformations. Glycoproteins were resolved by SDS-PAGE and were detected by immunoblot analysis probed with anti-6xHis antibody (red) and SBA lectin (green). Coordinates corresponding to position of clones in the 96 well plate are denoted on the blots.

To determine whether these refactored *pgl* loci displayed the *C. jejuni* heptasaccharide on their cell surface, individual clones were cultured in 96-well plates, washed to remove culture medium, and cell suspensions were transferred to a nitrocellulose membrane. Cell surface glycan production was assessed by probing spots with soybean agglutinin (SBA) lectin, which specifically binds terminal GalNAc residues within the *C. jejuni* heptasaccharide. Two independent replicates of the final assembly reaction and transformation were performed (Fig. 3, replicate 1, and Supplementary Fig. S1, replicate 2). We observed a number of clones that displayed crossreactivity with SBA, which indicate the biosynthesis and surface exposure of the GlcGalNAc_5_diNAcBac heptasaccharide (Fig. 3A). Furthermore, two clones from the first assembly and 1 clone from the second assembly appeared to exhibit greater fluorescent signal compared with cells containing the native unmodified cluster, suggesting superior glycan production. By contrast, we observed no signal in a control strain harboring an empty plasmid.

In this dot blot screen, cell suspensions were not normalised to the same optical density (OD), therefore, variation in the signal may be due to the differential cell growth and not altered cell surface glycan display. We selected 8 clones from the first replicate which displayed either high- or low- signal for further characterisation. These clones were cultured, their ODs (600 nm) were normalised, washed, and spotted on to nitrocellulose membrane as described above (Fig. 3B). Of these 8 clones, comparable signals between the two methods were observed with the exception of clones *pgl*1.H2 and *pgl*1.A5, which exhibited high signals in the initial 96-well plate screen but considerably lower qualitative signal when OD-matched.

To determine the relative abundance of the glycan moiety present on the cell surface, whole-cell ELISA was performed on the same 8 clones. A strain containing an empty plasmid was used to establish a baseline for OD readings. Clones *pgl*1.C7 and *pgl*1.C8 exhibited 2- and 1.75-fold increased cell surface-exposed GalNAc relative to the strain containing the unmodified *pgl* cluster, which suggested superior heptasaccharide production in these strains (Fig. 3C). Glycan-surface abundance was further assessed by densitometry analysis performed on the OD-matched dot blots. Quantification of the spot pixel intensities revealed a linear relationship between the fluorescent signal and the OD of culture for the strain containing the unmodified *pgl* cluster (Supplementary Figs. S2 and S3). We observed significantly increased anti-glycan signal in clones *pgl*1.C7, *pgl*1.C8, and *pgl*2.F6 (1.94-, 1.88-, and 1.77-fold, respectively) relative to those containing the native locus (Supplementary Fig. S4).

### Refactored *pgl* locus improves ExoA-heptasaccharide glycoconjugate yield in *E. coli*

By refactoring the *pgl* locus, the quantity of surface-displayed glycan could be improved in comparison to the native *pgl* locus. To determine whether these refactored loci would also improve the yield of glycoconjugate, the 8 clones previously selected were transformed into an *E. coli* strain that was specifically engineered as a glycoconjugate production platform. This chassis strain is derived from the *waaL*-, *wecA*+ strain, CLM24, and expresses a chromosomal copy of the *C. jejuni* OST *pglB* (Abouelhadid et al. 2021)*.* The carrier protein ExoA from *Pseudomonas aeruginosa* was encoded on a second plasmid and harbors two PglB glycosylation sequons. WecA is the initiator glycosyltransferase for the *E. coli* O-antigen and Enterobacterial common antigen synthesis pathways and catalyzes the transfer of GlcNAc onto UndP. Previous studies have shown that expression of the native *pgl* pathway in this strain results in the synthesis of a hybrid glycan that contains a mixture of GlcNac and diNAcBac at its reducing end (Wacker et al. 2006). ExoA was purified by His-affinity chromatography and was analyzed by western blotting using a protein-specific anti-His antibody and glycan-specific SBA lectin. Glycosylation of ExoA was observed in strains transformed with the variant plasmids *pgl*1.C7, *pgl*1.C8, and *pgl*2.F6 (Fig. 3D and E). Here, glycosylation efficiency was broadly comparable to strains transformed with the wild-type cluster. No glycosylation was observed in strains containing plasmids *pgl*1.A5 and *pgl1*.F6, which is consistent with the previous dot blot and ELISA experiments in which they produced less cell surface glycan. However, no glycosylation was also observed in the strains containing plasmids *pgl*2.A5, *pgl*2.B8, and *pgl*2.F12 despite these strains producing comparable surface glycan to *pgl*1.F6, as determined by ELISA.

**Fig. 4:**
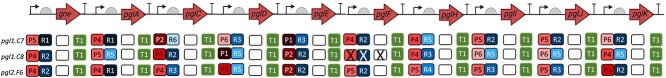
Sequencing of the three characterized *pgl* pathway variants revealed the genes:parts combinations for each gene in the *C. jejuni N*-glycosylation pathway. No promoter, no RBS, and a truncated gene product were detected for the *pglF* gene in locus *pgl*1.C8.

Sequencing of those plasmids which produced both LPS-linked and glycoconjugate glycans, *pgl*1.C7, *pgl*1.C8, and *pgl*2.F6 was undertaken and revealed a variety of combinations of promoter and RBS strengths corresponding to each CDS (Fig. 4). Plasmids *pgl*1.C7 and *pgl*2.F6 were found to have correct assembly of parts with intact CDS within the refactored loci. By contrast, sequencing of clone *pgl*1.C8 revealed that *pglF* had assembled incorrectly and contained a premature stop codon, resulting in a truncated gene product. *pglF* encodes a 4,6 UDP-GlcNAc dehydratase and is the first gene in the pathway for the synthesis of diNAcBac (Olivier et al. 2006). Any effect of this mutation is unlikely to be evident in the glycoproteins produced from chassis strains such as CLM24 or 10-β, where any deficiency in diNAcBac synthesis would be compensated by the endogenous *wecA* gene. Indeed, we observed negligible difference in the glycoconjugate yield between the *pglF* mutant pathway and correctly assembled loci.

**Fig. 5:**
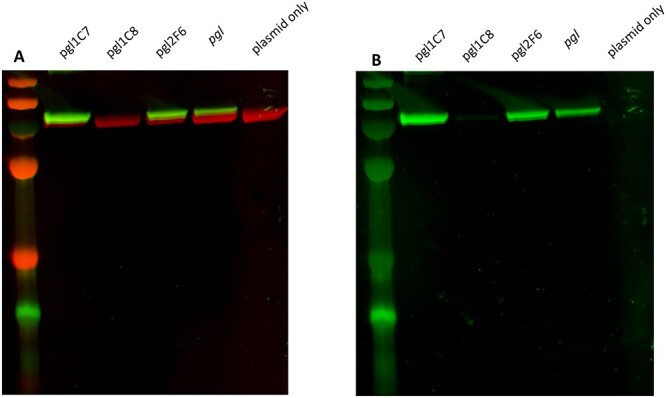
Glycoconjugate production by 3 refactored *pgl* cluster variants in the *wecA- waaL-* W3110-derivative SDB1. Hexahistidine tagged-ExoA with 2 potential glycosylation sequons was used as a substrate acceptor protein for PglB. A) Glycoproteins were resolved by SDS-PAGE and were detected by immunoblot analysis probed with anti-6xHis antibody (red) and SBA lectin (green). B) Anti-glycan SBA-only (green) channel.

The relaxed specificity of the PglA transferase, which recognizes both GlcNAc and diNAcBac as substrates, means that biosynthesis of the *C. jejuni* heptasaccharide in *wecA+* strains effectively bypasses the activity of PglC, PglD, PglE, and PglF enzymes, rendering them functionally redundant. Therefore, we investigated whether our refactored loci were able to synthesize and transfer diNAcBac and thus reproduce the native *C. jejuni* heptasaccharide as efficiently as the unmodified cluster. We transformed our refactored loci and plasmids encoding *exoA* and *pglB* into the *wecA-*, *waaL-*, W3110-derivative, SDB1, to determine glycoconjugate production. Resulting glycoproteins were purified, normalised to the same protein concentration, and analysed by western blotting as before (Fig. 5A). An increase in the anti-glycan signal was observed in resolved glycoproteins corresponding to the strains containing plasmids *pgl*1.C7 and *pgl*2.F6, which indicates an increase in the glycan:protein ratio and improved glycoconjugate yield in these strains. Interestingly, the production of a glycoconjugate was observed in the strain containing plasmid *pgl*1.C8 despite this pathway being possessing a premature stop codon in the diNAcBAc biosynthesis gene, *pglF*. However, the SBA signal was markedly reduced compared to glycoconjugates purified from cells containing the other *pgl* pathways, suggesting that glycan production is severely impaired in the absence of functional PglF. PglC orthologues have been shown to demonstrate a relaxed substrate specificity and can complement the function of WecA by transferring GlcNAc to UndP when heterologously overexpressed in vivo (Harding et al. 2018), which may explain the rescue of glycan production observed here.

### Refactored *pgl* locus improves sequon site occupancy of AcrATag-heptasaccharide glycoconjugate in *E. coli*

**Fig. 6:**
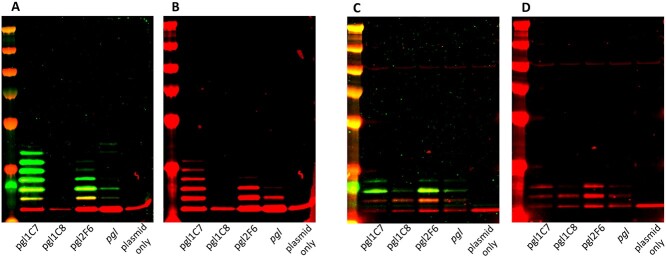
Glycoconjugate production by 3 refactored *pgl* cluster variants in the A and B) *wecA- waaL-* W3110-derivative SDB1 and the C and D) *wecA+ waaL- CLM24*. A modified version of Hexahistidine tagged-AcrA with 7 glycosylation sequons was used as a PglB substrate acceptor protein. Glycoproteins were resolved by SDS-PAGE and were detected by immunoblot analysis probed with SBA lectin (green) and anti-6xHis antibody (red). Merged SBA lectin (green) and anti-6xHis (red) channels are shown in panels A and C; anti-6xHis only channel is shown in panels B and D.

To determine whether glycosylation sequon site occupancy was altered in glycoconjugates derived from our refactored loci, a hepta-glycotag acceptor protein was constructed based on the *C. jejuni* protein AcrA. This acceptor protein was modified to contain 3 internal and 4 C-terminal PglB substrate sequons and was designated as AcrATag. *acrATag* was coexpressed with the refactored *pgl* clusters and the native *pgl* locus as a control in CLM24 and SDB1 chassis strains, and the resulting glycoconjugate production was assessed by western blotting as described above (Fig. 6). We observed hexa- and quinta- glycosylated AcrATag when it was coexpressed with plasmids *pgl*1.C7 and *pgl*2.F6, respectively, in the chassis strain SBD1 (Fig. 6A). While we did observe a heptaglycosylated species when AcrATag was coexpressed with the native *pgl* pathway, the overall SBA signal was reduced compared with the glycoconjugates derived from the *pgl*1.C7 and *pgl*2.F6 pathways, indicating an inferior glycoprotein yield. Crossreactivity with anti-His (the protein alone) suggested the production of quinta-, tri-, and di- glycosylated proteins resulting from coexpression with *pgl*1.C7, *pgl*2.F6, and the native locus, respectively (Fig. 6B). Relative glycan:protein ratio was assessed by sandwich ELISA (Supplementary Fig. S5) and densitometry analysis performed with 3 independent biological replicates (Supplementary Figs. S6 and S7), which revealed that AcrATag derived from cells with the *pgl*1.C7 or *pgl*2.F6 pathways was conjugated with 2.3- (ELISA) or 3.3-fold (densitometry) and 2.3- (ELISA) or 2.2-fold (densitometry) more glycan, respectively, relative to the protein purified from cells containing the native *pgl* locus. Consistent with previous observations, reduced glycoprotein production was detected in cells containing the *pgl*1.C8 pathway.

By contrast, qualitatively little difference in the overall glycoconjugate yields were observed when a *wecA*+ strain was used as the chassis (Fig. 6C and D). Triglycosylated protein species were detected in cells containing pathways *pgl*1.C7, *pgl*2.F6, and the native locus, as indicated by crossreactivity with SBA (Fig. 6C). Diglycosylated AcrATag was observed in cells containing pathway *pgl*1.C8, suggesting a relatively lower glycosylation efficiency. An increased SBA signal was observed in the diglycosylated AcrATag derived from pathways *pgl*1.C7 and *pgl*2.F6 compared to the unmodified locus. However, these differences were not as pronounced as those observed in glycoproteins produced from SDB1 (Fig. 6A). Interestingly, we observed relatively reduced glycosylation of AcrATag when a *wecA+* was used as a chassis strain compared with a *wecA-* strain. This is in contrast to the glycosylation of ExoA (Figs. 3D and 5), where we observed superior glycosylation in the *wecA+* chassis, and highlights the imperative to test each glycan-acceptor protein combination empirically to determine the optimal parameters for glycoconjugate production.

### Mass spectrometry confirms the structure of the glycan on ExoA

**Fig. 7:**
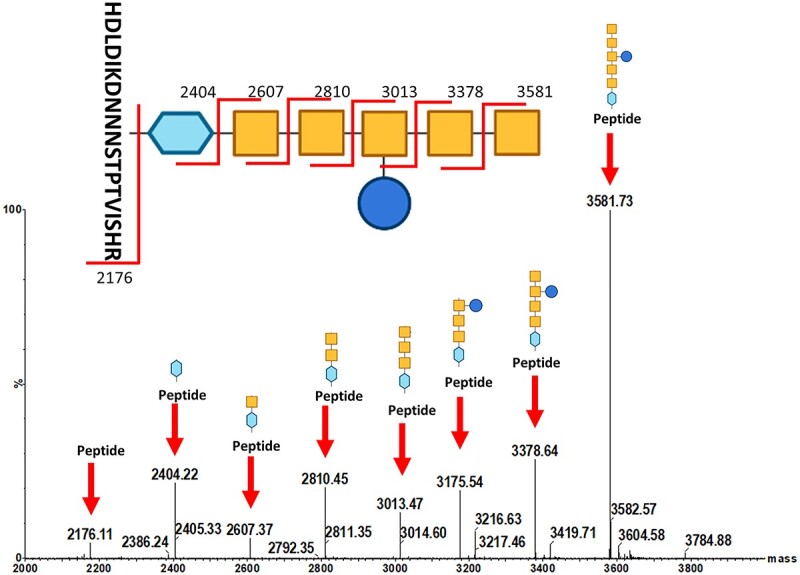
MS analysis of glycopeptides from ExoA derived from SDB1 (*wecA- waaL-*) with refactored *pgl* cluster clone, *pgl*1C7. Spectra were produced by fragmentation of the glycan structure-attached glycosylation sites digested by trypsin (DNNNS). Peaks indicative of fragmentation of the *N-*glycans are highlighted by red arrows. Blue squares, GlcNAc; blue hexagon, diNAcBac; yellow squares, GalNAc; blue circle, Glc.

To precisely determine the *N*-linked glycan covalently attached to ExoA, mass spectrometry analysis was undertaken. In-gel trypsin digestion was performed, and the resulting peptides were analyzed by liquid chromatography–tandem mass spectrometry (LC–MS/MS). ExoA was identified after the raw data were searched for a protein and a minimum of 1 peptide per protein as determined by the proteinlynx global server (PGLS). Further analysis was performed with the addition of the accurate mass of the *C. jejuni* heptasaccharide, GlcGalNAc_5_diNAcBac glycan (C_56_H_91_N_7_O_34_), to the modification list in Biopharmlynx. This analysis revealed a doubly charged peptide (HDLDIKDNNNSTPTVISHR) that was modified with the expected glycan mass (Fig. 7). The corresponding parental peak (m/z 3581) and the fragmentation peaks were analyzed using Masslynx and showed the sequential loss of 203 Da (HexNAc) and 162 Da (Hex), confirming that the expected heptasaccharide was attached to ExoA. Furthermore, we observed a peak of m/z 2404, which is consistent with the expected mass-to-charge of diNAcBac (m/z 228) attached to the peptide (m/z 2176). We observed no peaks corresponding to HexNAc attached directly to the peptide (expected m/z 2379), which indicates that diNAcBAc was the reducing end sugar. Overall, these data confirm the faithful reproduction of the native heptasaccharide from refactored cluster *pgl*1.C7 and its transfer to the carrier protein ExoA by PglB.

## Discussion

Despite the glycan moiety being the critical antigenic component of a glycoconjugate vaccine, relatively few attempts have been made to improve glycan yield by altering the biosynthetic locus itself. This is partly because “en bloc” cloning and transfer of the unmodified gene cluster can result in “adequate” glycan production, which is sufficient for the engineering of a prototype glycoconjugate vaccine. Recent developments in synthetic biology provide a tractable alternative to “en bloc” cloning by using DNA assembly for the bottom-up construction of redesigned clusters from individual parts. In this study, we employ such a synthetic biology approach to the assembly of the archetypal bacterial *N-*linked *C. jejuni* glycosylation locus in *E. coli*, identifying synthetic variants with improved yield of glycan and glycoconjugate production relative to the native unmodified pathway following a single combinatorial round of assembly without iterative optimisation.

In recent years, a number of strategies have been employed to rationally modify bacterial cells used for the production of glycoconjugates in a process known as “chassis engineering.” Varying flux through the phosphotransferase system (Pandhal et al. 2013), glyoxylate cycle (Pandhal et al. 2011), or blocking glycolysis and the pentose phosphate pathway (Wayman et al. 2019; Jiang et al. 2022) have been shown to improve the overall glycoconjugate yield. Similarly, modifying pathways for precursor sugar biosynthesis can result in an increased glycan and glycoconjugate yield (Pandhal et al. 2012; Kay et al. 2022). Typically, these modifications are made to the chromosome of the chassis *E. coli* strains and require successive targeted deletion (or introduction) of individual genes. These approaches often require significant *a priori* knowledge of the endogenous and incoming glycan metabolic pathways and how they may conflict. By targeting the glycan biosynthesis locus with an unbiased, empirical approach, we make minimal assumptions of the existing endogenous and incoming pathways, which makes this system ideally suited for the engineering of relatively poorly characterised glycan structures. Furthermore, the regulatory networks that drive glycan biosynthetic loci are rarely investigated, and it is frequently assumed that they have a polycistronic configuration driven by a single promoter. Such a configuration may not necessarily be optimal for the heterologous glycan production in a non-native host strain. Reconstructing the locus by systematically varying the expression level of each enzyme allows the rapid identification of which pathway variants perform well by screening. Our data show that after screening a library of <200 clones, we were able to identify an expression profile for each gene within the *pgl* pathway which outperformed that of the native locus. Conceivably, these assembly methods can also be combined with the rationally designed methods described above to further improve glycoconjugate yield.

Chassis engineering of the host strain by introducing PGCT components onto the chromosome is another strategy employed to improve glycoconjugate yield. For example, integration of a codon optimized, single copy of *pglB* into the chromosome of strain CLM24 (*waaL-*) resulted in the increased glycosylation of the acceptor protein with *C. jejuni* heptasaccharide, albeit at a cost of decreased overall glycoconjugate yield (Strutton et al. 2017). Expression of heterologous genes from the chromosome rather than multicopy plasmids is a well-recognised strategy for alleviating the toxic effects of metabolically burdensome enzymes, such as PglB, which resides in the cytoplasmic membrane (Bentley et al. 1990; Bassalo et al. 2016; Englaender et al. 2017). However, in a recent study, Terra et al. integrated the entire unmodified *pgl* pathway onto the chromosome of the chassis strain SDB1 (*wecA*- and *waaL*-; Terra et al. 2022) and observed greater glycoconjugate yield in 3 out of 4 acceptor proteins tested when an additional inducible copy of *pglB* was also provided on a plasmid. Optimal glycosylation was achieved when both the glycan biosynthesis genes and *pglB* were encoded on a plasmid in their native configuration. This suggests that the stoichiometry and relative expression level of each enzyme in the pathway influenced the conjugation efficiency. In our study, we observed superior glycosylation of AcrATag when *pglB* was encoded on a plasmid and was expressed in a *wecA-* strain compared with *pglB* on the chromosome in a *wecA+* strain. Taken together, these data suggest that there is no one-size-fits-all strategy for the engineering of glycoconjugate vaccines and highlights the necessity to empirically test optimal production parameters. In this study, we focused on optimising glycan biosynthesis rather than conjugation per se. However, our data demonstrate that superior glycosylation efficiency was achieved when our refactored pathways were combined with both chromosomally and plasmid-encoded *pglB*. Combinatorial DNA assembly could also be applied to identify suitable expression levels of the oligosaccharyltransferase and acceptor protein to further improve glycoconjugate yield.

Improving yield of glycan biosynthesis has advantages beyond protein-based glycoconjugates. Expression of bacterial glycans in hypervesiculating strains of *E. coli* offers an alternative approach to protein-based glycoconjugates. Many Gram-negative bacteria produce outer membrane vesicles (OMVs), which are composed of lipopolysaccharide, outer membrane, and periplasmic proteins. Several vesicle-based based vaccines against meningococcal disease have already been developed and are licensed in many countries (Bjune et al. 1991) and there are other bacterial OMV vaccine candidates currently in development (Roberts et al. 2008; Schild et al. 2009; McConnell et al. 2011). However, current methods for producing these vaccines involve isolation of OMVs directly from the pathogenic organism of interest. By exploiting similar pathways for glycan surface display presented in this study, *E. coli* can be engineered to decorate lipid A with recombinant glycans from other pathogenic bacteria. Purification of OMVs from such laboratory strains of *E. coli* offers a safe, cheap alternative toward the production of these vaccines (Price et al. 2016).

In summary, we refactored the model *C. jejuni pgl* cluster using Start-Stop Assembly, constructed a combinatorial library of cluster variants, and demonstrated faithful glycan production and transfer to an acceptor protein by PglB. Despite screening a small fraction of the maximum number of possible clones within the library, we identified clones that not only produce the glycan or glycoconjugate but even outperform glycan production from the unmodified *C. jejuni pgl* cluster. Overall, these results demonstrate the potential of this synthetic biology approach toward an improved heterologous glycan biosynthesis and subsequent glycoconjugate vaccine production.

## Materials and methods

### Strains and plasmids

Tables of strains and plasmids used in this study are shown in Supplementary Tables S1 and S2, respectively. The *E. coli* strains were cultured on LB agar and LB broth at 37 °C. Antibiotics were added as necessary at the following concentrations: tetracycline 20 μg mL^−1^, ampicillin 100 μg mL^−1^, kanamycin 50 μg mL^−1^, and chloramphenicol 30 μg mL^−1^.

### Construction of the PglB substrate protein, AcrAtag

The AcrAtag acceptor protein is a modified and truncated version of the *C. jejuni* lipoprotein, AcrA (amino acids 61 to 211). Amino acids 98–113 and 151–165, which flank the native AcrA PglB glycosylation sequon at Asn123, were deleted as these have previously been shown to not be required for glycosylation of Asn123. Two additional internal sequons at Asn117 and Asn147 were introduced through mutation at Phe115Asp and Thr145Asp, respectively, and 4 glycosylation sequons were added as C-terminal glycotags, which were separated by glycine-arginine-glycine linkers. Mutations at amino acids Lys97Gln and Lys191Gln were made to reduce proteolytic cleavage. An N-terminal DsbA signal sequence was included for trafficking the protein to the periplasm and a C-terminal hexahistidine tag was included for protein purification. The “acrAtag” construct was ordered for synthesis as a g-block (IDT) and was inserted into pEC415 using Gibson assembly using primers* acrAtag*_pEC415_f and r and the pEC415 backbone amplified using pEC415_*acrAtag *f and r. Amplification using Phusion polymerase and Gibson assembly using the NEB HiFi kit were both performed according to the manufacturer’s instruction.

### Construction of refactored *C. jejuni N-*linked protein glycosylation locus (*pgl*) libraries

Individual parts, expression units, and biosynthetic pathways were constructed by a hierarchical Start-Stop Assembly as previously described (Taylor et al. 2019). Genetic parts were cloned in the level 0 storage plasmid pStA0 by mixing the PCR amplified or chemically synthesized fragments in a reaction with T4 DNA ligase buffer, 20 U/μL of T4 DNA ligase, and 0.5 U/μL of BsaI. The reaction mixtures were incubated in a thermocycler for 30 cycles of 37 °C for 5 min and 16 °C for 5 min before a single final step of deactivation at 65 °C for 20 min. Ligated fragments were transformed into *E. coli* 10β by electroporation and were selected on LB agar supplemented with ampicillin. Level 0 mixtures of 6 different promoters and 6 different RBSs (described previously, Taylor et al. 2019) were prepared as equimolar mixtures of the plasmids containing these parts. Libraries of level 1 plasmids (each containing whole expression units comprising a promoter, an RBS, a functional gene, and a transcriptional terminator) were obtained by Start-Stop Assembly reactions. For each functional gene, the reaction was performed in a total reaction volume of 20 μL in 1 × T4 DNA ligase buffer and included 20 fmol of the destination vector (pStA1AB, pStA1BC, pStA1CD, pStA1DE, or pStA1EZ), 40 fmol of the mixture of Level 0 plasmids carrying promoters, 40 fmol of the mixture of Level 0 plasmids carrying RBSs, 40 fmol of Level 0 plasmid carrying the corresponding gene, and 40 fmol of Level 0 plasmid carrying a terminator. Level 1 library reactions were performed as above, and transformants were selected on LB agar-supplemented tetracycline. Plasmid DNA was isolated from the resulting libraries by miniprep, pooled, and used in subsequent level 2 reactions. The level 2 library was generated with similar Start-Stop Assembly reactions, but the BsaI restriction enzyme was used instead of SapI and transformants were selected on LB agar supplemented with Kanamycin. Plasmids DNA was isolated from the resulting libraries as above and final level 3 libraries (which encode the full glycan biosynthetic pathway) were generated with the Start-Stop assembly reactions with BbsI restriction enzyme and selection on chloramphenicol.

### Dot blot screening of refactored *pgl* biosynthetic pathways

Single-colony transformants were picked with a sterile toothpick and grown in 200 μL LB supplemented with chloramphenicol in 96-well polystyrene plates (Nunc) shaking at 600 rpm at 37 °C overnight. Cells were subcultured to fresh LB and chloramphenicol and were grown to stationary phase. Cultures were sedimented by centrifugation (2,800 × *g*, 10 min) and were washed with 300 μL of sterile phosphate buffered saline (PBS). Cell suspensions were sedimented and were washed 2 further times (3 total), and cells were resuspended in 150 μL of sterile PBS.

For low-throughput, OD-matched dot blots, cells were grown in 5 mL of LB supplemented with chloramphenicol shaking at 200 rpm at 37 °C overnight. Cultures were normalized to OD600 nm = 5 and were sedimented by centrifugation (6,000 × *g*, 10 min). Pellets were washed 3 times with sterile PBS.

Cell suspensions (2.5 μL) were spotted onto nitrocellulose membrane and were air dried for 30 min. Membranes were washed with PBS containing 0.1% (v/v) Tween 20 (PBST) and were probed with Biotinylated SBA lectin (Vector Laboratories) at a 1 in 10,000 dilution for 45 min. Membranes were washed 3 times with PBST and were incubated with Streptavidin IRDye 800CW (LI-COR Biosciences) at a dilution of 1 in 10,000 in PBST for 40 min. Fluorescent signal was detected with the Odyssey LI-COR detection system (LI-COR Biosciences).

### Glycoconjugate expression and purification

Plasmids encoding refactored *pgl* loci were transformed into respective chassis strains by electroporation and were selected on LB containing appropriate antibiotics. Strains were cultured in LB broth at 200 rpm at 37 °C to early exponential phase upon which expression was induced by the addition of 1 mM Isopropyl β-D-1-thiogalactopyranoside (IPTG, Sigma) and 0.2% (w/v) L-arabinose (Sigma). Cultures were incubated for a further 16 h at 37 °C, sedimented by centrifugation, and resuspended in 50 mM Tris HCl pH 8, 300 mM NaCl, 10 mM imidazole. Cells were lysed using BeadBug zirconium lysing tubes (Sigma) in a FastPrep homogenizer (MP Biomedicals) and insoluble material was removed by pelleting. Hexahistidine tagged proteins were subsequently purified from cell lysates by Ni-NTA affinity chromatography and were eluted in 50 mM Tris HCl pH 8, 300 mM NaCl, and 300 mM imidazole.

### Immunoblot analysis

Protein concentration was determined by Bradford assay (BioRad), samples were loaded with an equal protein content, resolved by SDS-PAGE, and transferred to nitrocellulose membranes using the iBlot 2 dry blotting system (ThermoFisher Scientific). Membranes were probed for the presence of GalNAc containing moieties as above. His-tag containing proteins were probed using mouse anti-His monoclonal primary antibody (ThermoFisher Scientific) and goat antimouse IgG IRDye 680 (LI-COR Biosciences). Fluorescent signal was detected with the Odyssey LI-COR detection system (LI-COR Biosciences).

### Whole-cell ELISA

Semiquantitative analyses of surface-exposed glycan matched by OD were determined by ELISA. Cell cultures were grown overnight, washed 3 times in carbonate buffer (15 mM Na_2_CO_3_ and 35 mM NaHCO_3_) and were normalized to OD600nm = 1. Transparent polystyrol 96-well plates with high protein-binding capacity (F96 MaxiSorp, Nunc) were coated with OD-matched cell suspensions (50 μL per well) and were incubated overnight at 4 °C. Wells were then washed 4 times with 300 μL PBST (0.1% Tween-20) and were blocked for 1 h at room temperature with 200 μL in PBST per well, shaking at 500 rpm. After blocking, wells were washed 2 times with 300 μL PBST per well. To quantify surface-exposed terminal GalNAc, biotin-conjugated SBA lectin (Vector Laboratories) was added to each well at a 1:10,000 dilution in PBST (100 μL per well). The plate was incubated for 1 h at room temperature, 500 rpm, and unbound lectin was removed by washing the wells 4 times with 300 μL PBST per well. Streptavidin-HRP (Invitrogen) was added at 1:10,000 dilution in PBST (100 μL per well), the plate was incubated for 1 h at room temperature, and unbound antibodies were removed by 4 washes. ELISA plates were developed with 100 μL per well tetramethylbenzidine (TMB) substrate solution (Invitrogen). The oxidative reaction was stopped by adding 100 μL per well H_2_SO_4_ (2 N), and ODs at 450 nm were determined using a SpectraMax iD5 plate reader (Molecular Devices). OD450nm background values (buffer only in wells treated with biotinylated-SBA lectin and streptavidin-HRP) were subtracted from test values. Technical and biological triplicates were averaged, values represent the arithmetic mean, and error bars represent the standard deviations of biological triplicates.

### Glycoconjugate sandwich ELISA

Glycoconjugates were purified as described above; concentrations were quantified using the Qubit Protein assay (Thermo Fisher Scientific) on a Qubit 2.0 instrument as per the manufacturer’s instructions. Relative glycan abundance was quantified by sandwich ELISA as previously described (Mauri et al. 2021) with minor modifications. Briefly, Polystyrene 96-well plates (F96 MaxiSorp, Nunc) were coated with mouse anti-His monocolonal antibody (Thermo Fisher Scientific), diluted 1:1,000 in PBS, and were incubated overnight at 4 °C. Wells were washed 4 times with PBST and were blocked with Carbo-free blocking buffer (Vector Laboratories) for 30 min at room temperature. Glycoconjugates were normalized to the lowest measured concentration of protein (234 ng μL^−1^) and were then diluted 1:50. Unbound glycoconjugate was removed by washing with PBST. Wells were incubated with biotin-conjugated SBA in a 1:1,000 dilution in PBST for 30 min at room temperature. Unbound lectin was removed by washing with PBST. Wells were then incubated with Streptavidin-HRP (Invitrogen) at 1:10,000 dilution in PBST for 30 min and unbound Streptavidin was removed as before. Plates were developed by adding 100 μL of TMB substrate solution (Invitrogen) per well, following which the reaction was stopped by addition of 100 μL H_2_SO_4_ (2 N). ODs (450 nm) were determined using a SpectraMax iD5 plate reader. Data were transformed using log10 to approximate a normal distribution and then regression analysis was used to determine the significant differences in glycan production between strains.

### Densitometry of purified glycoconjugates

Glycoconjugates were purified, resolved by SDS-PAGE, and immunoblotted as described as above. Fluorescent signal was detected with the Odyssey LI-COR detection system, images were converted to greyscale, and quantification of signal intensity was performed using ImageJ software (v1.53t). A rectangle with defined area was used to gate around each band corresponding to a single glycosylated protein species within each gel lane. The same area rectangle was also used to quantify the background by taking a measurement from an empty lane. Data were transformed using log10 to approximate a normal distribution and then regression analysis was used to determine the significant differences in glycan production between strains.

### Mass spectrometry analysis

In gel reduction, alkylation, and digestion with trypsin or chymotrypsin were performed on the gel sample prior to subsequent analysis by mass spectrometry. Cysteine residues were reduced with dithiothreitol and were derivatized by treatment with iodoacetamide to form stable carbamidomethyl derivatives. Trypsin digestion was carried out overnight at room temperature after initial incubation at 37 °C for 2 h.

Sample digests, resuspended in 0.1% (v/v) formic acid, were analyzed by online nano-flow reverse-phase high-performance LC with online electrospray-MS analysis with MS/MS (MSe) using a Waters SYNAPT G2-S high-definition mass spectrometer, which was coupled to a Waters ACQUITY UPLC M-Class System (Waters UK, Elstree). Separations were achieved by means of a C18 trapping column (M-Class Symmetry C18 Trap, 100 Å, 5 μm, 180 μm × 20 mm, 2G) connected in line with a 75-μm C18 reverse-phase analytical column (M-Class Peptide BEH C18, 130 Å, 1.7 μm, 75 μm × 150 mm), eluted over 90 min with a gradient of acetonitrile in 0.1% formic acid at a flow rate of 300 nL/min. Column temperatures were maintained at 50 °C, and data were recorded in MSe “Resolution”-positive ion mode, with scan times set to 0.5 s in both the high-energy and low-energy modes of operation. The instrument was precalibrated using 10–100 fmol/μL of [Glu1]-fibrinopeptide B/5% (v/v) acetic acid (1:3, v/v) and was calibrated during analysis by means of a lockmass system using [Glu1]-Fibrinopeptide B 785.84262+ ion. The collision gas utilized was argon with collision energy ramp of 20–45 eV. Data acquisition was performed using MassLynx (Waters UK, Elstree) software and data were analyzed by means of MassLynx, BiopharmaLynx and PLGS version 3.0.2 (Waters UK, Elstree).

## Supplementary Material

SupplementaryFiguresandTables_cwac082

supfiguresfinal_cwac082
