# Supplementary material for: A combinatorial DNA assembly approach to biosynthesis of N-linked glycans in E. coli
Source: Glycobiology. 2023 Jan 13;33(2):138–49. doi: 10.1093/glycob/cwac082 (PMC9990991; doi:10.1093/glycob/cwac082)
Supplement: SupplementaryFiguresandTables_cwac082 [file supplementaryfiguresandtables_cwac082.docx]

**Supplementary Figure legends**

**Figure S1**

**A** Randomly-picked clones probed for production of the *C. jejuni* heptasaccharide using a Soy Bean Agglutinin (SBA), a biotinylated GalNAc specific lectin and green streptavidin-coupled IRDye. Clones were selected at random, grown in high throughput 200μl cultures and spotted on a nitrocellulose membrane. Clones selected for further characterisation are circled in red. **B** Clones were subsequently grown in 10ml cultures, normalised to the same optical density and probed for GalNAc production with SBA.

Cells containing refactored loci from the first screen are denoted “1.C7” and “1.C8”. Cells harbouring plasmid-borne native unmodified *pgl* cluster and empty plasmid control are denoted “+” and “-” respectively.

**Figure S2**

Cells harbouring the native *pgl* pathway (pglΔpglB), variant pathway clones *pgl*1.C7, *pgl*1.C8 and *pgl*2.F6 and a plasmid only control were grown overnight in LB, washed and normalised to the following optical densities (OD600nm) 10, 7.5, 5, 2.5, 1 and 0.5 for native *pgl* pathway or 5, 2.5 and 1 for the variant pathways. Cell suspensions were spotted on a nitrocellulose membrane and production of GalNAc was determined by binding with SBA. Three independent cultures were grown, diluted and spotted for each strain.

**Figure S3**

Cell suspensions harbouring the native *pgl* pathway (pglΔpglB) were washed and normalised to optical densities (OD600nm) 10, 7.5, 5, 2.5, 1 and 0.5 were spotted on a nitrocellulose membrane and probed for production of GalNAc by binding with SBA (see Figure S4). Fluorescent signal was detected with the Odyssey LI-COR detection system, spots were converted to greyscale, and signal intensity was quantified using ImageJ. Signal intensity (Arbitrary units, AU) was plotted against optical density. Biological triplicates were averaged, values represent the arithmetic mean and error bars represent standard deviations of biological triplicates.

**Figure S4**

Cells harbouring the native *pgl* pathway (pglΔpglB) and variant pathway clones *pgl*1.C7, *pgl*1.C8 and *pgl*2.F6 were grown overnight LB, washed, normalised to OD600= 2.5 and spotted on a nitrocellulose membrane (see Figure S2). Production of GalNAc was determined by binding with SBA, fluorescent signal was detected using the LI-COR detection system and spot signal intensity was quantified using ImageJ. Data from biological triplicates was transformed using log10, to approximate a normal distribution, and then regression analysis was used to determine significant differences in glycan production between strains (*** *p*<0.001, ** *p*<0.01). Error bars represent standard deviation of the mean.

**Figure S5**

Glycosylation of the acceptor protein AcrATag with the *C. jejuni* heptasaccharide from cells (SDB1, *wecA waaL*) harbouring the native *pgl* pathway (pglΔpglB), variant pathway clones *pgl*1.C7, *pgl*1.C8 and *pgl*2.F6 and a plasmid only control (pGT415) was determined by sandwich ELISA. Glycoproteins were normalised to the same concentration of protein, were bound to a polystyrene plate and glycan abundance was determined by binding to SBA lectin. Data from biological triplicates was transformed using log10, to approximate a normal distribution, and then regression analysis was used to determine significant differences in glycan production between strains (* *p*<0.05). Error bars represent standard deviation of the mean.

**Figure S6**

Glycosylation of the acceptor protein AcrATag with the *C. jejuni* heptasaccharide from cells (SDB1, *wecA waaL*) harbouring the native *pgl* pathway (pglΔpglB), variant pathway clones *pgl*1.C7, *pgl*1.C8 and *pgl*2.F6 and a plasmid only control (pGT415) was determined by Western blot. Glycoproteins were His-affinity purified, resolved by SDS-PAGE and probed with anti-His antibody (red) and SBA lectin (green). Three independent biological replicates were performed for each strain.

**Figure S7**

Densitometry analysis of glycosylated AcrATag modified with the *C. jejuni* heptasaccharide from cells (SDB1, *wecA waaL*) harbouring the native *pgl* pathway (pglΔpglB), variant pathway clones *pgl*1.C7, *pgl*1.C8 and *pgl*2.F6 and a plasmid only control (pGT415). Glycoproteins were purified, analysed by Western blot (Figure S6) and intensity of SBA signal was quantified by ImageJ. Signal intensity (Arbitrary Units, AU) is plotted on the *y* axis. Data from biological triplicates was transformed using log10, to approximate a normal distribution, and then regression analysis was used to determine significant differences in glycan production between strains (*** *p*<0.001, * *p*<0.05). Error bars represent standard deviation of the mean.

**Supplementary Tables**

**Table SI, Bacterial strains used in this study**

| **Strains** | **Genotype** | **Reference** |
| --- | --- | --- |
| *E. coli* 10β | F^–^ *endA1* *deoR*^+^ *recA1* *galE15* *galK16* *nupG* *rpsL* Δ*(lac)X74* φ80*lacZΔM15* *araD139* Δ*(ara,leu)7697* *mcrA* Δ*(mrr-hsdRMS-mcrBC)* Str^R^ λ^–^ | New England Biolabs |
| *E. coli* W3110 | F- *mcrA mcrB* In(*rrnD-rrnE*)1 | ATCC 27325 |
| *E. coli* *cedA::pglB* | CLM24 ΔwaaL, *cedA::pglB* | (Abouelhadid et al., 2021) |
| *E. coli* CLM37 | W3110 Δ*wecA* | (Linton et al., 2005) |
| *E. coli* SDB1 | W3110 ΔwaaL, ΔwecA | (Garcia-Quintanilla et al., 2014) |

**Table SII, Plasmids used in this study**

| **Plasmid** | **Description** | **Source** |
| --- | --- | --- |
| pACYC184 *pglpglB::kan* | Native *C. jejuni pgl* cluster with inactivated *pglB* | (Linton et al., 2002) |
| pEC415*acrAtag* | Modified version of *C. jejuni* *acrA* that includes seven N-glycosylation sequons | This study |
| pSTA0 | Start-Stop Assembly empty storage vector | (Taylor et al., 2019) |
| pSTA1AB | Start-Stop Assembly Level 1 vector (A and B fusion sites) | (Taylor et al., 2019) |
| pSTA1BC | Start-Stop Assembly Level 1 vector (B and C fusion sites) | (Taylor et al., 2019) |
| pSTA1CD | Start-Stop Assembly Level 1 vector (C and D fusion sites) | (Taylor et al., 2019) |
| pSTA1DE | Start-Stop Assembly Level 1 vector (D and E fusion sites) | (Taylor et al., 2019) |
| pSTA1EZ | Start-Stop Assembly Level 1 vector (E and Z fusion sites) | (Taylor et al., 2019) |
| pSTA212 | Start-Stop Assembly Level 2 vector (1 and 2 fusion sites) | (Taylor et al., 2019) |
| pSTA223 | Start-Stop Assembly Level 2 vector (2 and 3 fusion sites) | (Taylor et al., 2019) |
| pSTA313 | Start-Stop Assembly Level 3 vector (1 and 3 fusion sites) | (Taylor et al., 2019) |
| p*pgl*1.C7 | *pgl* pathway clone | This study |
| p*pgl*1.C8 | *pgl* pathway clone | This study |
| p*pgl*2.F6 | *pgl* pathway clone | This study |

**Table SIII, Oligonucleotides used in this study**

| **Oligonucleotide** | **Description** | **Sequence** |
| --- | --- | --- |
| OligoGT234 | pStA0 Fw sequencing primer | GGGGAAACGCCTGGTATCT |
| OligoGT235 | pStA0 Rv sequencing primer | AGCAAAAACAGGAAGGCAAA |
| OligoGT339 | pStA1 Fw sequencing primer | GTTGAGGACCCGGCTAGG |
| OligoGT340 | pStA1 Rv sequencing primer | TGTGACGGAAGATCACTTCG |
| gneseq | *gne* sequencing primer | TTACTACGCCCATAAGGATTAA |
| pglAseq | *pglA* sequencing primer | GCGTGTATTCATCTTGCGGC |
| pglCDseq | *pglCD* sequencing primer | TCATATTTAGGCAAGGTGTTTTCA |
| pglEseq | *pglE* sequencing primer | CTCCAAATTCTCCAAAAGTTCCTAA |
| pglFseq | *pglF* sequencing primer | ATCGAAGATGCTGCTGAAGCT |
| pglHseq | *pglH* sequencing primer | TGTGCGACTCTGCGAAAAGAA |
| pglIseq | *pglI* sequencing primer | CTTGGAAGCAGTGATAAGGTGT |
| pglJseq | *pglJ* sequencing primer | AGGCAATAAAGTCGCCACAA |
| pglKseq | *pglK* sequencing primer | CTATGCTTGAAGACGATAAATTAAG |
| acrAtagpEC415f | Fw primer for amplification of *acrAtag for insertion into pEC415* | GAATAACATAATGAAAAAAATCTGGTTAGCTT |
| acrAtagpEC415r | Rv primer for amplification of *acrAtag for insertion into pEC415* | TCAAGAATTCTTAGTGGTGGTGATGGTG |
| pEC415acrAtagf | Fw primer for amplification of pEC415 backbone for insertion of *acrAtag* | CCACCACTAAGAATTCTTGAAGACGAAAGG |
| pEC415acrAtagr | Rv primer for amplification of pEC415 backbone for insertion of *acrAtag* | AGATTTTTTTCATTATGTTATTCCTCCTTATTTAAAATG |
| Lv0gneFwd | Fw primer for amplification of *gne* and cloning into pStA0 | AAGGGGTTGGTCTCATGTGGCTCTTCGATGAAAATTCTTATTAGCGGTGGTG |
| Lv0gneRev | Rv primer for amplification of *gne* and cloning into pStA0 | CAGTGTTGGGTCTCTGGTCGCTCTTCATTAACACTGTTTTTCCCAATCAAAAG |
| Lv0pglAFwd | Fw primer for amplification of *pglA* and cloning into pStA0 | AAGGGGTTGGTCTCATGTGGCTCTTCGATGAGAATAGGATTTTTATCACATG |
| Lv0pglARev | Rv primer for amplification of *pglA* and cloning into pStA0 | CAGTGTTGGGTCTCTGGTCGCTCTTCATTATACATTCTTAATTACCCTATCATA |
| Lv0pglEFwd | Fw primer for amplification of *pglE* and cloning into pStA0 | AAGGGGTTGGTCTCATGTGGCTCTTCGATGAGATTTTTTCTTTCTCCTCCG |
| Lv0pglERev | Rv primer for amplification of *pglE* and cloning into pStA0 | CAGTGTTGGGTCTCTGGTCGCTCTTCATTAAGCCTTTATGCTCTTTAAGATCAG |
| Lv0pglHFwd | Fw primer for amplification of *pglH* and cloning into pStA0 | AAGGGGTTGGTCTCATGTGGCTCTTCGATGATGAAAATAAGCTTTATTATCGCAAC |
| Lv0pglHRev | Rv primer for amplification of *pglH* and cloning into pStA0 | CAGTGTTGGGTCTCTGGTCGCTCTTCATTGGCATTTTTAACCTCGGCTATAAG |
| Lv0pglIFwd | Fw primer for amplification of *pglI* and cloning into pStA0 | AAGGGGTTGGTCTCATGTGGCTCTTCGATGCCTAAACTTTCTGTTATAGTACC |
| Lv0pglIRev | Rv primer for amplification of *pglI* and cloning into pStA0 | CAGTGTTGGGTCTCTGGTCGCTCTTCATTATTTTTGCATAAAGCCACCCGAATT |
| Lv0pglJFwd | Fw primer for amplification of *pglJ* and cloning into pStA0 | AAGGGGTTGGTCTCATGTGGCTCTTCGATGCAAAAATTAGGCATTTTTATTTATTC |
| Lv0pglJRev | Rv primer for amplification of *pglJ* and cloning into pStA0 | CAGTGTTGGGTCTCTGGTCGCTCTTCATTTCCTAATAAATATTTCAAAGCATC |
| Lv0pglKFwd | Fw primer for amplification of *pglk* and cloning into pStA0 | AAGGGGTTGGTCTCATGTGGCTCTTCGATGTTAAAAAAACTTTTTTTTATTTTAAGTAA |
| Lv0pglKRev | Rv primer for amplification of *pglk* and cloning into pStA0 | CAGTGTTGGGTCTCTGGTCGCTCTTCATTTTTCTCCTCTTTAAGCTTACCG |

**List of synthetic DNA sequences used in this study**

**CjpglC**

GGTCTCATGTGGCTCTTCGATGTACGAAAAAGTATTTAAGCGCATCTTCGATTTCATCTTAGCTCTGGTACTTTTGGTGCTTTTCTCTCCTGTCATCCTTATCACAGCATTACTTTTGAAAATTACACAGGGTAGTGTCATCTTCACCCAGAATCGCCCAGGGTTGGATGAAAAGATCTTCAAAATTTACAAGTTTAAGACTATGTCGGACGAGCGCGATGAGAAGGGGGAGTTATTATCCGATGAACTTCGCCTTAAAGCCTTCGGTAAGATTGTTCGCTCCTTGAGCCTGGACGAATTGCTGCAATTATTTAACGTTCTGAAGGGGGACATGTCCTTCGTGGGTCCTCGTCCTTTGCTTGTCGAGTACTTGTCTTTATATAACGAGGAGCAAAAATTACGTCACAAGGTCCGTCCGGGTATTACTGGGTGGGCCCAGGTAAATGGTCGCAACGCAATCTCGTGGCAGAAGAAGTTTGAATTAGACGTCTATTACGTGAAGAATATTAGTTTCCTTTTAGATTTGAAGATCATGTTTCTGACTGCGCTGAAGGTTCTGAAACGCAGTGGAGTGTCCAAAGAGGGCCACGTGACTACTGAAAAGTTCAATGGGAAGAATTAATGAAGAGCGACCAGAGACC

**CjpglD**

AAGGGGTTGGTCTCATGTGGCTCTTCGATGGCAAGAACTGAAAAAATTTATATTTATGGTGCTAGTGGTCATGGGCTTGTTTGTGAAGATGTGGCTAAAAATATGGGCTATAAAGAATGTATTTTTTTAGATGATTTTAAAGGAATGAAATTTGAAAACACCTTGCCTAAATATGATTTTTTTATAGCTATAGGAAACAATGAAATTCGAAAAAAGATTTATCAAAAAATTTCAGAAAATGGCTTTAAAATAGTTAATCTTATTCATAAAAGTGCACTTATAAGTCCTAGTGCAAGCGTGGAAGAAAATGCAGGGATTTTAATTATGCCCTATGTAGTGATTAACGCTAAAGCCAAAATAGAAAAAGGCGTGATTTTAAATACTTCAAGTGTGATTGAGCATGAATGCGTGATAGGGGAATTTTCTCATGTAAGCGTAGGGGCTAAATGTGCGGGTAATGTAAAAATCGGTAAAAATTGTTTTTTAGGGATTAATTCTTGTGTTTTGCCTAATTTAAGCTTAGCAGATGATAGTATTTTGGGTGGTGGAGCAACTTTGGTTAAAAGCCAAAATGAAAAAGGTGTTTTTGTGGGAGTGCCTGCAAAAAGAAAAATATAATGAAGAGCGACCAGAGACCCAACACTG

**CjpglF**

GGTCTCATGTGGCTCTTCGATGATCTTCTACAAATCTAAACGTCTGGCGTTTTTCCTCATATCTGACATCGTTCTGATCCTGCTGTCTGTTTACCTGGCGTTCTCTCTGCGTTTCTCTGGTGACATCCCGTCTATCTTCTACCACGGTATGATGGTTTCTGCGATCATCCTGCTGGTTCTGAAACTGTCTTTCCTGTTCGTTTTCCGTATCTACAAAGTAGCTTGGAGATTTTTTTCTCTCAATGAAGCAAGAAAAATTTTTATCGCTTTGCTTTTAGCTGAATTCTGCTTCTTCCTGATCTTCTACTTCTTCTCTGACTTCTTCAACCCGTTCCCGCGTTCTGCGATCGTTATCGACTTCGTTCTGTCTTACATGTTCATCGGTACCCTGCGTATCTCTAAACGTATGCTGGTTGACTTCAAACCGTCTCGTATGAAAGAAGAAGAAACCCCGTGCATCGTTGTTGGTGCGACCTCTAAAGCGCTGCACCTGCTGAAAGGTGCGAAAGAAGGTTCTCTGGGTCTGTTCCCGGTTGGTGTTGTTGACGCGCGTAAAGAACTGATCGGTACCTACTGCGACAAATTCATCGTTGAAGAAAAAGAAAAAATCAAATCTTACGCGGAACAGGGTGTTAAAACCGCGATCATCGCGCTGCGTCTGGAACAGGAAGAACTGAAAAAACTGTTCGAAGAACTGGTTGCGTACGGTATCTGCGACGTTAAAATCTTCTCTTTCACCCGTAACGAAGCGCGTGACATCTCTATCGAGGACCTGCTGGCGCGTAAACCGAAAGACCTGGACGACTCTGCGGTTGCGGCGTTCCTGAAAGACAAAGTTGTTCTGGTTTCTGGTGCGGGTGGTACCATCGGTTCTGAACTGTGCAAACAGTGCATCAAATTCGGTGCGAAACACCTGATCATGGTTGACCACTCTGAATACAACCTGTACAAAATCAACGACGACCTGAACCTGTACAAAGAAAAAATCACCCCGATCCTGCTGTCTATCCTGGACAAACAGTCTCTGGACGAAGTTCTGAAAACCTACAAACCGGAACTGATCCTGCACGCGGCGGCGTACAAACACGTTCCGCTGTGCGAACAGAACCCGCACTCTGCGGTTATCAACAACATCCTGGGTACCAAAATCCTGTGCGACTCTGCGAAAGAAAACAAAGTTGCGAAATTCGTTATGATCTCTACCGACAAAGCGGTTCGTCCGACCAACATCATGGGTTGCACCAAACGTGTTTGCGAACTGTACACCCTGTCTATGTCTGACGAAAACTTCGAAGTTGCGTGCGTTCGTTTCGGTAACGTTCTGGGTTCTTCTGGTTCTGTTATCCCGAAATTCAAAGCGCAGATCGCGAACAACGAACCGCTGACCCTGACCCACCCGGACATCGTTCGTTACTTCATGCTGGTTGCGGAAGCGGTTCAGCTGGTTCTGCAGGCGGGTGCGATCGCGAAAGGTGGTGAACTGTTCGTTCTGGACATGGGTAAACCGGTTAAAATCATCGACCTGGCGAAAAAAATGCTGCTGCTGTCTAACCGTAACGACCTGGAAATCAAAATCACCGGTCTGCGTAAAGGTGAAAAACTGTACGAAGAACTGCTGATCGACGAAAACGACGCGAAAACCCAGTACGAATCTATCTTCGTTGCGAAAAACGAAAAAGTTGACCTGAACTGGCTGAACAAAGAAATCGAAAACCTGCAGATCTGCGAAGATATCTCTGAAGCGCTGCTGAAAATCGTTCCGGAATTCAAACACAACAAAGAAGGTGTTTAATGAAGAGCGACCAGAGACC
