# Supplementary figures and images for: A combinatorial DNA assembly approach to biosynthesis of N-linked glycans in E. coli
Source: Glycobiology. 2023 Jan 13;33(2):138–49. doi: 10.1093/glycob/cwac082 (PMC9990991; doi:10.1093/glycob/cwac082)

**Figure S1**

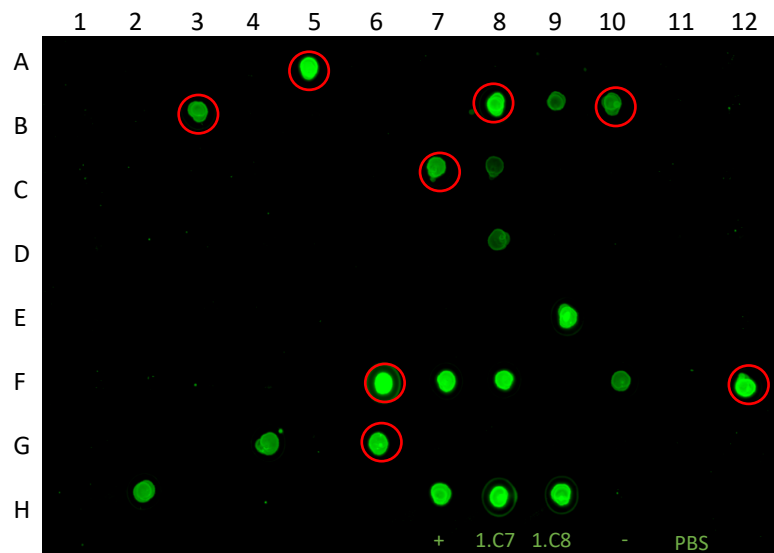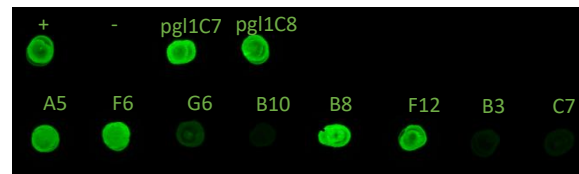

Figure S2

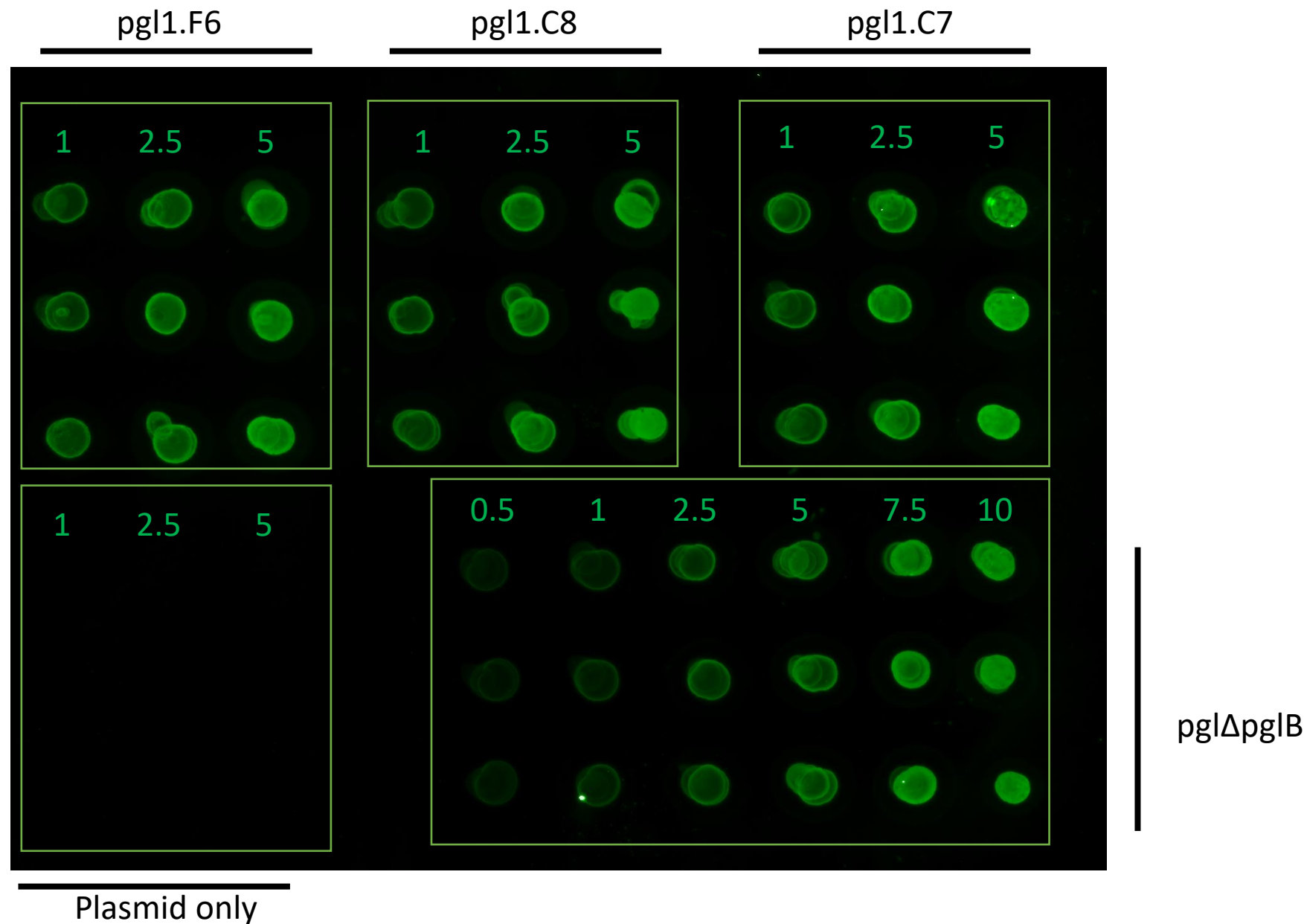

Figure S3

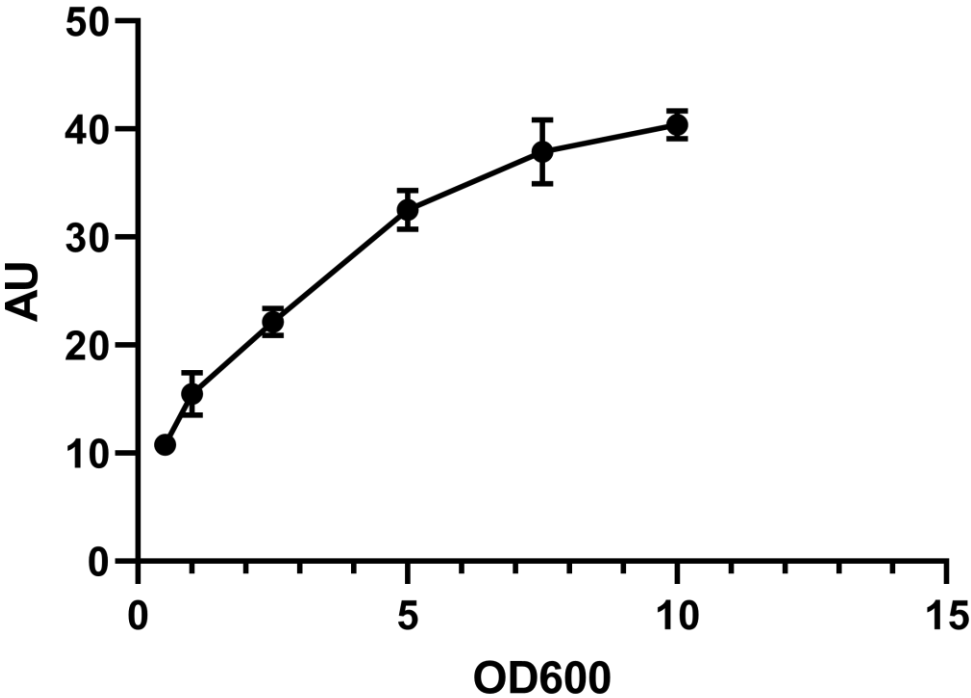

Figure S4

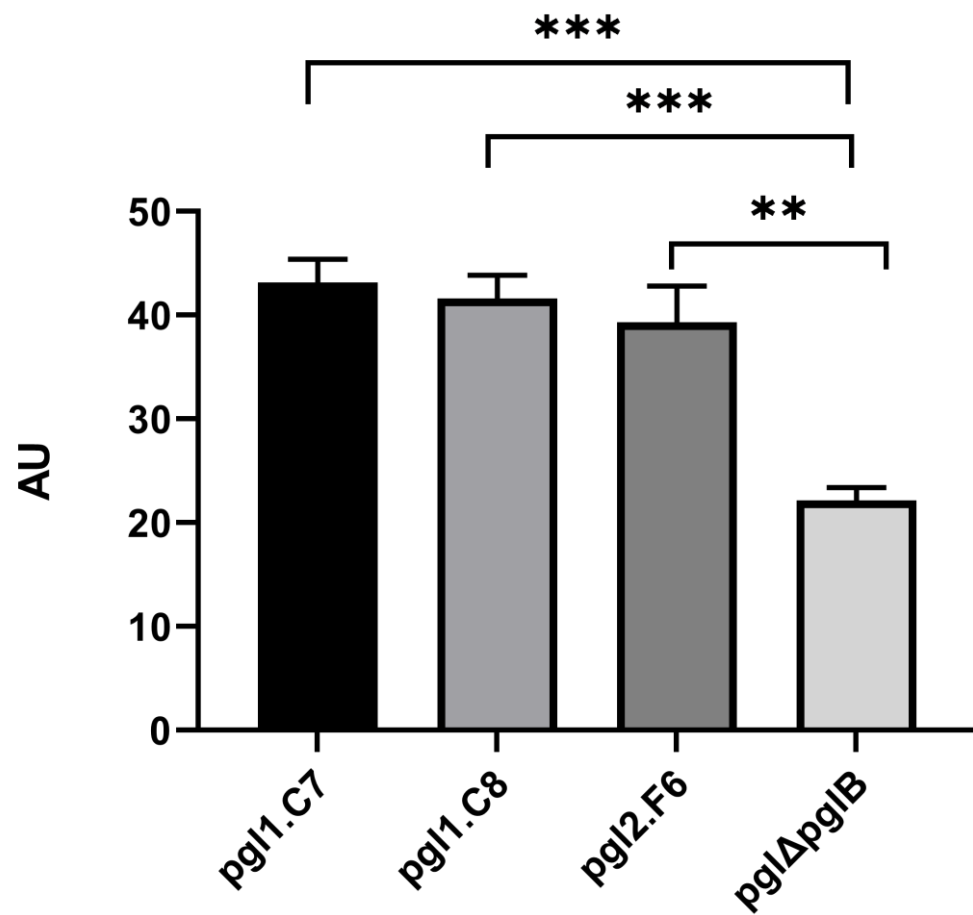

Figure S5

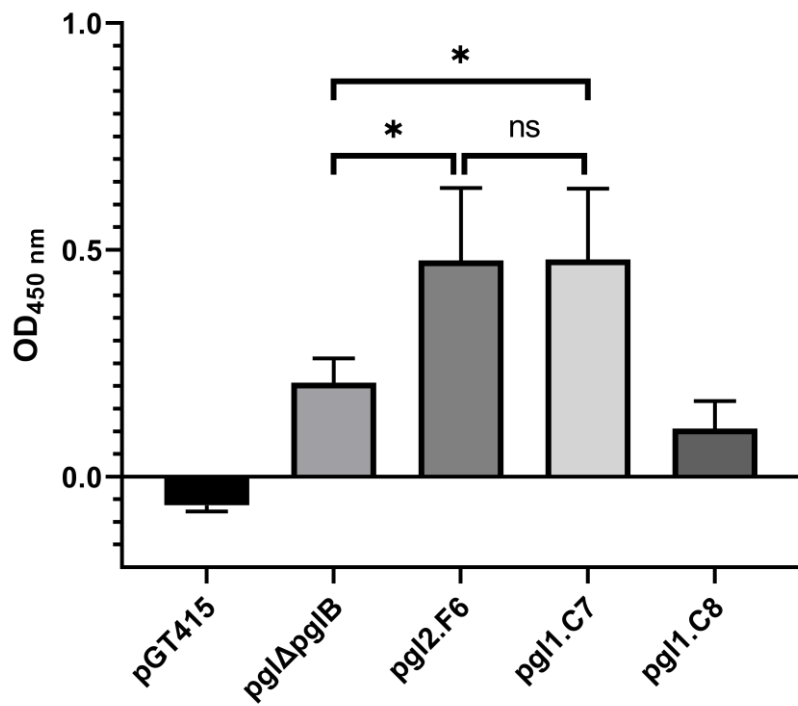

Figure S6

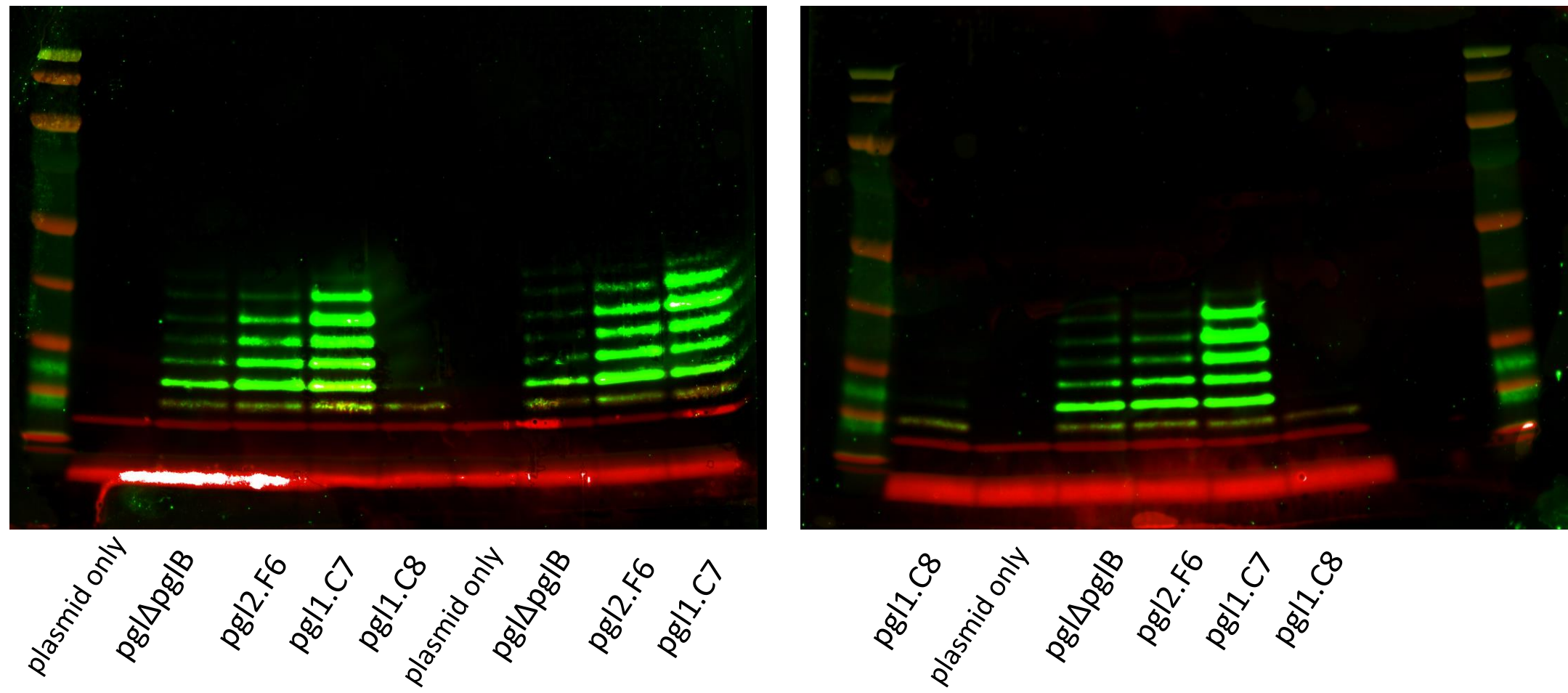

Figure S7

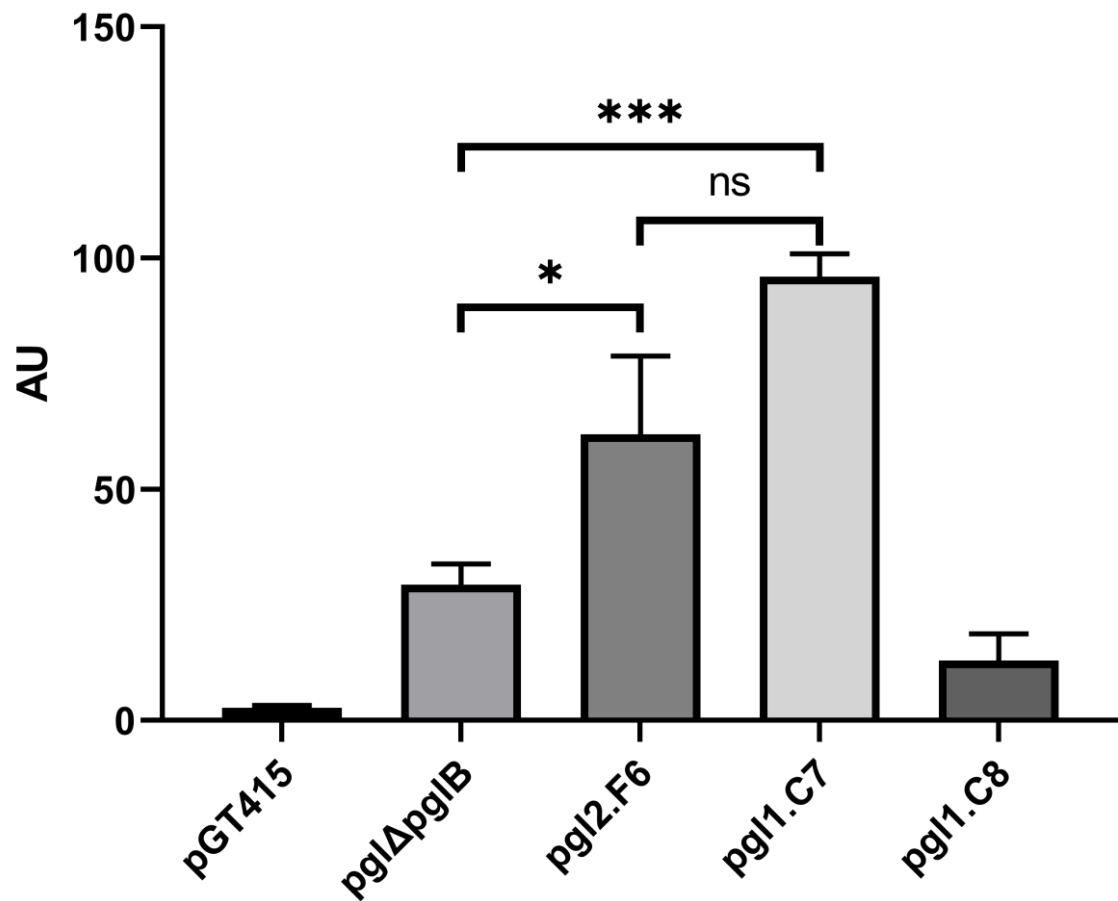

Supplement: supfiguresfinal_cwac082 [file supfiguresfinal_cwac082.pdf]
